# News packaging during a pandemic: A computational analysis of news diffusion via Facebook

**DOI:** 10.1177/17504813231177280

**Published:** 2023-06-09

**Authors:** Jonathan Hendrickx, Annelien Van Remoortere, Michael Opgenhaffen

**Affiliations:** University of Vienna, Belgium; Universiteit van Amsterdam, The Netherlands; KU Leuven, Belgium

**Keywords:** Computational analysis, Facebook, journalism, news diffusion, polarity, social media, subjectivity

## Abstract

Facebook remains the most important platform where social media editors package and try to ‘sell’ media outlets’ online news articles to audiences. In one of the first studies of its kind, we assess how this practice was effectuated during the first year of the COVID-19 pandemic. We use computational analysis to determine the polarity, subjectivity and use of some linguistics features in the status messages of 140,359 Facebook posts of 17 mainstream and alternative news titles from Flanders (Belgium) between March 2020 and 2021. Among other things, we find that status messages score considerably higher than headlines in terms of polarity and subjectivity, and that they, along with the use of question and interrogation marks, peaked in the first months of the pandemic. We contextualise our findings within existing scholarship and wider trends in increasingly digitised and globalised media societies.

## Introduction

The COVID-19 pandemic continues to dramatically alter multiple facets of ordinary lives. This also applies to news and journalism, where the crisis has exacerbated the already ongoing dramatic disruption within newsrooms and journalism as a profession. Online news consumption skyrocketed at the onset of the pandemic, with heightened importance for social media platforms as diffusion gateways to generate online traffic for news titles’ websites (Newman et al., 2021). News organisations have hired designated social media editors to promote the online distribution of their news items (Welbers and Opgenhaffen, 2018) by repackaging and effectively trying to ‘sell’ online articles to increase traffic to news brands’ websites (Opgenhaffen, 2021).

In this paper, we collected CrowdTangle data to assess all Facebook posts of 17 mainstream and alternative news media outlets from Flanders (Belgium) that were published between 1 March 2020 and 1 March 2021 (*n* = 140,359). Through computational analysis, we measure the tonality (in terms of subjectivity and polarity), use of emoji and linguistics features (e.g. question and exclamation marks) of the status messages as written by social media editors that accompany news articles as shared on Facebook by building on previously published research (e.g. Hågvar, 2019; Haim et al., 2021; Welbers and Opgenhaffen, 2019). We differentiate posts per (type of) outlet, as well as between pandemic-related articles and other types of news. This multi-faceted approach allows us to combine three distinct emerging topics of media-related research: COVID-19, social media editors and platformisation.

## COVID-19 and (digital) journalism

### News production

In their inquiry of French-speaking Belgian journalists, Libert et al. (2021) denoted ‘a sharp contrast between challenging working conditions (isolation, lack of expertise and job losses in worst cases) and the satisfaction that comes from the social contribution of their reporting’ (p. 1). Perreault and Perrault (2021) go as far as suggesting that the COVID-19 pandemic ‘presents a gap between journalistic understanding and journalism practice’ (p. 986) as journalists themselves experienced effects of the pandemic on their personal lives, while trying to inform the citizenry as robustly and completely as possible.

In seeking to compare differences in the way news packaging occurs for news related and not related to the COVID-19 pandemic, we do not necessarily assume that such differences exist. Rather, we aim to contribute to the notion of reporting on media hypes. Vasterman (2005) developed a classification system for how a key event and subsequent similar incidents, isolated or compared to the key event, can fuel both media-generated (e.g. opinion pieces, background articles or marginal references) or source-generated fractions through action or reaction. This leads to a ‘well-known paradox that the more action society takes the more visible the problem will get, reinforcing social concern’ (p. 526). This also ties in with the insights regarding the so-called hype pipeline (Caulfield and Condit, 2012). This concept refers to the fact that when interest in science news grows – for example, in the case of a pandemic – there are numerous actors and factors that exert pressure to make the news more spectacular, which in turn creates more interest from the public for it. Which in turn creates more pressure from the various actors, and so on. By translating this framework to the contemporary newsroom setting in which designated social media editors (SMEs) play important roles to package and ‘sell’ news content via social media platforms, we shed additional light on how this was effectuated in the first year of the pandemic.

### News content

News content, as the obvious result of production, too has been altered by the pandemic, and just a few studies have already assessed this empirically at the time of writing. Hendrickx and Van Remoortere (2021) found that news content was shared considerably more between two leading Belgian newspapers during the first months of the pandemic when compared to preceding time frames, with negative effects on content and overall news diversity. In a cross-border study, which is slightly more aligned with our own study, Mellado et al. (2021) studied the use of sources in pandemic-related reporting of 78 mainstream news outlets from Brazil, Chile, Germany, Mexico, Spain, the United Kingdom and the United States. They analysed the actors and sources in nearly one million Facebook, Twitter and Instagram posts throughout 2020. As opposed to previous studies on health-related news, the authors found a dominance for political sources in social media reporting, particularly in Latin American nations. They note that ‘(a) pandemic of this magnitude is in this sense similar to a war; it is a kind of social context in which the role of the state in society is strongly enhanced’ (p. 20). This ties in with a quantitative content analysis on actors in Flemish television news broadcasts in 2020 (Walgrave and Kuypers, 2021). This analysis revealed that politicians of ruling parties were much more present as actors than their colleagues of opposition parties, which was argued to be negative for actor, source and overall content diversity within mainstream news content.

In a working paper, Quandt et al. (2020) executed a sentiment analysis of Facebook posts of 110 German mainstream and alternative news outlets on COVID-19-related and other types of news. They found that alternative titles were considerably more negative in their pandemic-related reporting as opposed to legacy brands, and that the differences were ‘extremely significant’ (p. 19). Masullo et al. (2022) compared the content of Facebook posts of American local newspapers and television news channels with a survey of citizens. They revealed that there was a big discrepancy between the public interest in COVID-19-related news and what was reported on, with more interest in fact-checks but more economic and business news delivered. In both aforementioned cases, the data set only included news titles’ Facebook posts of the very beginning of the pandemic. We contribute to scholarship by using a similar approach to quantitatively assess the polarity and subjectivity of status messages of Facebook posts (including their use of emoji and linguistics features) of Belgian news media throughout the first year of the COVID-19 pandemic, as will be further outlined in next sections of the paper at hand.

## The role and responsibility of social media editors

### Different logics

The murky position of a social media editor (SME) and their duties within newsrooms complicates labelling their output with a specific term. We do not refer to Facebook status messages accompanying links to online news articles as news content in the strict sense of the word, previously defined as ‘pieces of information created for an intended target audience with the prime objective of informing it as objectively as possible on current affairs in one specific field’ (Hendrickx and Ranaivoson, 2019: 4). Hence, we do not claim to study news content, but rather the specific, engagement-oriented type of social media content intended to ‘sell’ online news articles online and to increase news content’s consumption which is an outcome of news production. The typical output of a SME on platforms such as Facebook perfectly corresponds with the four grounding principles of social media logic as theorised by van Dijck and Poell (2013). Programmability relates to links to both technology and the relevance of human agency, which in our case is exerted by SMEs. The second principle is popularity, which the authors argue is ‘conditioned by both algorithmic and socio-economic components’ (p. 7) and is an obvious key factor in determining the success of the work of SMEs. Typically, this is expressed in audience engagement data such as likes, shares and comments on platforms such as Facebook. Connectivity, or combining human connectedness and automated personalisation, is the third principle and applicable to SMEs. Datafication, finally, also plays a crucial role in social media logic and thereby for the work of SMEs, particularly with the added dimension of real-time data availability.

Various studies have signalled marketing functions becoming endemic in contemporary newsrooms, including the role of SMEs as ‘marketeers’ of news content across online platforms which harms the traditional gatekeeping role of mass media (Neilson and Gibson, 2021; Tandoc and Vos, 2016). This ties in with the evolution of audience participation and engagement having become firmly embedded in all aspects of the news production process and journalistic practices (Loosen, 2019). Scholarship has discussed the existing friction between the social media logic of more engagement driven news values and editing on one hand, and mass media logic entailing traditional journalistic standards and news brand characteristics on the other. Early studies in the field shone light on the newness of SMEs and the lack of direction or strategy to foster content distribution and audience participation (Hille and Bakker, 2013). In her dissertation on the role of social media editors in American newsrooms, DeVito (2014) concluded that fellow journalists perceive them as ‘social media gurus’, and that ‘social media sites are not replications of websites, and audience members want to see and read about stories that are not necessarily news’ (pp. 75–76).

Siegert et al. (2011) suggested that the role of social media as a gateway platform and the advent of designated SMEs within newsrooms has elevated the commodification of news titles as news brands, which defines how journalists edit and process news and sets new journalistic standards. This distinct social media logic within a professional news context has been argued to be ‘informed in large part by technological, economic, and professional affordances’ (Haim et al., 2021, p. 410). In her analysis of SMEs in Switzerland and Finland, Lischka (2021) questioned ‘whether a media system aiming at serving the informational needs of its citizens can sufficiently serve social media users in the context of engagement-rewarding algorithms’ (p. 444). In a similar study of Israeli peers, Tsuriel et al. (2021) highlight how SMEs ‘connect and negotiate between (. . .) two media logics’ (p. 1988), mass and social media logics and create their own hybrid media logics and structures.

### The element of surprise (and emotion)

In integrating social media platforms as content diffusion channels, media corporations and newsrooms have been forced to adapt to their many contingencies and constraints. All major news organisations have invested significantly in social media platforms, regardless of funding model or the type(s) of media they publish and/or broadcast (Sehl et al., 2021). As the most popular platform for news diffusion, Facebook’s algorithm prioritises emotion-provoking content, be it positive or negative. Surprising, entertaining and emotional news are three of the best ingredients to predict the success of a news title’s Facebook post, and are more prone to be liked and shared, or even to go viral (Harcup and O’neill, 2017). Surprise and emotions play a role not only in terms of the selection of topics, but also in the packaging of the news. On Facebook, for example, the status messages at the top of a post play an important role in capitalizing on the emotions of the social media news user. Expressing emotions in Facebook status messages to enhance Facebook engagement is one of the five strategies found by Hågvar (2019) in her study of Norwegian news media. The others are adding emojis, asking a question (‘Do you agree?’), posing a request (‘Tag your friends!’) and stating subjective points of view – the latter is particularly relevant for this study as we use computational analysis to measure the subjectivity of Facebook status messages of news organisations and to gauge whether subjectivity is effectively present, and to what extent. Along the same lines, Facebook status messages and their relevance for news publishers, have previously been defined by Welbers and Opgenhaffen (2019: 51):

‘Like headlines in newspapers, the status messages are placed at the top of the article and concisely refer to the article below. The message can be a word, a sentence, a small paragraph or a quote. Sometimes they resemble classic headlines by summarizing the core content of the news article, but they also take less conventional forms, such as joking about the news item, addressing the reader (e.g., ‘what do you think?’), or expressing clear emotions, possibly accompanied by one or multiple emoticons.’

The role and responsibility of social media editors in newsrooms was gauged in a series of 22 expert interviews across Belgian and Dutch media companies by Opgenhaffen (2021). In line with previously discussed scholarship, he notes that the editors frequently address people more directly via the Facebook status updates using activating or spoken language and subjectivity, ‘even if they prefer not to use the latter term because it would violate the principle of objectivity’ (Opgenhaffen, 2021: 135). The two main established aims are clear: increasing online traffic to the news outlets’ websites and fostering user engagement (e.g. likes, comments and shares) on platforms themselves. Other studies, which also relied on interviews with social media editors from various countries, only confirmed that emotional, surprising and/or subjective story elements are routinely employed to defy Facebook’s black box algorithm (Lischka, 2021; Tsuriel et al., 2021). The use of and appeal to user’s emotions through social media texts is a pinnacle in the argument of Wahl-Jorgensen (2020) that across all aspects of the news process a so-called ‘emotional turn’ is taking place.

Haim et al. (2021) analysed over one million posts from 699 active Scandinavian news outlets’ Facebook pages of 2018 and 2019. They find that calls to action through question marks and exclamation points as well as emoji are regularly applied to increase engagement and traffic, and that this differs from the more conventional headlines that are part of the online news article shared on Facebook. Welbers and Opgenhaffen (2019) specifically assessed the subjectivity and polarity of Facebook status messages and found that these status messages differ strongly from the more conventional headlines from the online news article, especially in the case of subjective elements in popular news media (pp. 56, 57). Headlines too are often modified when an article is transferred from the news site to Facebook, like in the case of COVID-19 when SMEs leave out words that bring nuance (Verstappen et al., 2022). The research by Lamot et al. (2022) showed that headlines are regularly adapted, and that adjustments such as adding emotional words can have a positive effect on engagement.

Based on the foregoing, there are enough arguments to believe that SMEs will use certain rhetorical techniques to sell the news in times of COVID-19. After all, this is a global news hype which makes the competition even greater than usual as every news medium around the world is reporting on the same topic. SMEs use techniques on Facebook to get noticed by the news user and the algorithm. We have seen that SMEs see the status message as an ideal way to do this. A news element where they can be a bit looser with the use of tonalities and linguistic features, it is not really part of the news report itself but rather part of the packaging of the news. However, specifically for pandemic-related news, we might suppose that this is less evident, since SMEs are somewhat more cautious about using rhetorical tricks to elicit engagement in the case of delicate or controversial news topics (Hågvar, 2019; Opgenhaffen, 2021). Especially in the early stages of the virus outbreak, reporting mostly focused on the number of deaths and patients in hospital ICUs, when neutral wording is arguably more appropriate. Since there are arguments for and against the use of these rhetorical strategies in status updates around COVID news, we formulate the following research question:

RQ1: What is the difference in terms of tonality, use of emoji and linguistics features in Facebook status messages of COVID-19-related news posts versus other news topics?

## Alternative media

Hiaeshutter-Rice and Weeks (2021) hypothesised that news outlets’ posts ‘that receive the most user engagement are often the most ideologically extreme’ (p. 518). To further contribute to scholarship, we seek to compare the output of SMEs on Facebook pages of alternative news outlets with that of mainstream titles. Larsson (2019) assessed the Facebook user engagement rates of Norwegian national, regional and hyperpartisan news titles. He found that while the latter outlets trailed in terms of raw follower numbers, the followers were much more active than with other types of news brands, which aided in amplifying their news content. In line with previous scholarship, the author linked this to a more outspoken mobilising tone in the Facebook status messages accompanying and ‘selling’ news content.

In the thus far only study which already assessed the relationship between alternative news outlets and COVID-19-related reporting, Boberg et al. (2020) concluded that German alternative news outlets’ pandemic coverage adheres to ‘their worldview and familiar narratives, primarily offering a populist, anti-systemic, anti-establishment perspective’ (p. 10). This study only dealt with news stories published in the first weeks after the proper outbreak of the pandemic in the Western hemisphere, in March 2021, when lockdowns were imposed, and public life came to a sudden standstill. We extend the scope by focusing on the status messages of Facebook posts of both mainstream and alternative news media from March 2020 to 2021, to shed more light on this unique relationship. The literature study and the revealed gaps in existing scholarships culminate in the second research question:

RQ2: What is the difference in terms of tonality, use of emoji and linguistics features in Facebook status messages of mainstream and alternative Flemish news brands?

## Study design

For this study, we collected Facebook posts via CrowdTangle, a tool that Facebook collaborated with until the summer of 2021. Specifically, we collected all Facebook posts from the 17 most well-known news media in Flanders (Belgium’s Dutch-speaking region) for 1 year, between March 1, 2020, the beginning of the COVID-19 pandemic in Belgium, and March 1, 2021, when the vaccination campaign was in full swing, and the pandemic lost momentum. This set of news media includes the eight traditional daily newspaper and magazine titles, two TV channels (of which one is public and one commercial) and seven online-only or so-called alternative news titles (of which three are distinctly left-wing oriented and two are right-wing). The outlets were chosen based on their presence and activity on Facebook in line of previous research (Hendrickx and Van Remoortere, 2021) and by constituting a diverse breadth of neutral, left-wing and right-wing news outlets. The full list of brands is available from the authors upon reasonable request. From these accounts, we collected all Facebook posts published in this constructed year (*n* = 140,359). To better pinpoint changes over time, as the pandemic gradually lost its novelty and became a fixture in everyone’s lives, we divided our findings over four smaller periods of 3 months each (see Table 1). These roughly overlap with the pandemic’s dominance in public life and debate from a Belgian perspective, although we find that it is also applicable and appropriate for international, though still predominantly Western-oriented research. March to May 2020 mark the period of the harshest lockdowns and restrictions, with eases and relaxations between June and August. Between September and November, infections and restrictions alike rose again. December to February 2021, finally, saw a continuation of most restrictions, including the holiday period and the advent of countries’ vaccination drives.

**Table 1.** Four clusters of status messages for non-COVID-19-related posts (*n* = 90,009) and COVID-19-related news posts (*n* = 50,350).

	Polarity (−1 to 1)	Subjectivity (0–1)	Total (%)
Neutral messages			
Non-COVID-19-related posts	−0.00	0.02	42,993 (47.7)
COVID-19-related posts	−0.01	0.02	24,126 (47.9)
Subjective messages, low polarity			
Non-COVID-19-related posts	0.08	0.50	24,306 (27,0)
COVID-19-related posts	0.07	0.48	15,645 (31.1)
Very subjective messages, outspoken positive			
Non-COVID-19-related posts	0.54	0.83	12,055 (13.4)
COVID-19-related posts	0.50	0.80	5481 (10.9)
Very subjective messages, outspoken negative			
Non-COVID-19-related posts	−0.40	0.77	10,655 (11.8)
COVID-19-related posts	−0.39	0.76	5098 (10.1)

We distinguished between COVID-19 news and other news using an automated dictionary approach with a self-created topic list (see Appendix A for the full list). To assemble this topic list, we browsed over articles and made a list of all frequently used or specific COVID-19 words used by journalists. After considering which words would be used regularly and mostly in COVID-19 related news coverage, we ended up with a list of 31 keywords (see Appendix A). Using a dictionary-based approach, messages containing any of these words were retained. After the automated topic coding was completed, we took a random sample of 100 Facebook posts and manually coded them. In 86% of the posts the topic coding was accurate.

For both the status updates and the more conventional headlines, we measured the degree of subjectivity and polarity. In doing so, we align with previous research (Welbers and Opgenhaffen, 2019) on language use in status updates. This was done by using the subjectivity lexicon for Dutch adjectives developed by De Smedt and Daelemans (2012). The lexicon is specifically aimed at measuring the amount of subjectivity and polarity. For subjectivity, words in the lexicon are given a value between zero and one, for polarity this ranges from negative (−1) to neutral (0) to positive (1). The lexicon is based on manually annotated frequently used adjectives (1100) and expanded to 5500 words using established machine learning techniques for dictionary expansion. The lexicon was tested on book reviews and generated an accuracy of 82%, with a precision of 0.80, a recall of 0.86 and an *F*1 score of 0.83.

In addition, we also measured whether the status updates contained emoji and thereby connect to previous research on this news element (Guo and Sun, 2022; Hågvar, 2019; Haim et al., 2021; Welbers and Opgenhaffen, 2019). In terms of linguistic features, we focus on the use of question marks and exclamation points since previous research (e.g. Haim et al., 2021; Lamot et al., 2022) has shown that these are key elements that can be strategically deployed in status updates and headlines on Facebook to elicit interaction. We did this by simply conducting a word search on these characters. By doing this for both status updates and conventional headlines, we can make the comparison between these two elements. Finally, we divided our 12-month period into four periods of 3 months to describe the evolution of Facebook messages through the first corona year.

## Results

**Figure 1.**
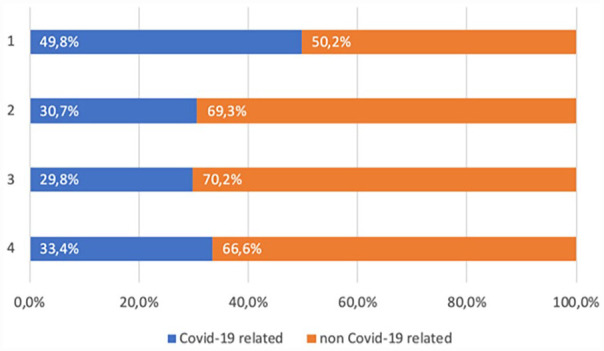
Share of pandemic and non-pandemic-related news as shared on Facebook over four 3 month-periods.

Of our total dataset of 140,359 Facebook news posts of Flemish news brands between 1 March 2020 and 2021, 50,350 or 35.87% of those dealt with the COVID-19 pandemic, meaning that 90,009 (64.13%) of the posts shared on Facebook were not related to the pandemic. The possible assumption that most news shared on Facebook in the first year of the pandemic was also related to it thus does not hold, at least not for the Flemish context. When we divide our findings per quarter, we see that at the start of the pandemic the percentage share of COVID-19 related news articles shared on Facebook dropped progressively after our first period of analysis before increasing again the fourth and final period, as shown in Figure 1.

### Status messages versus headlines

**Figure 2.**
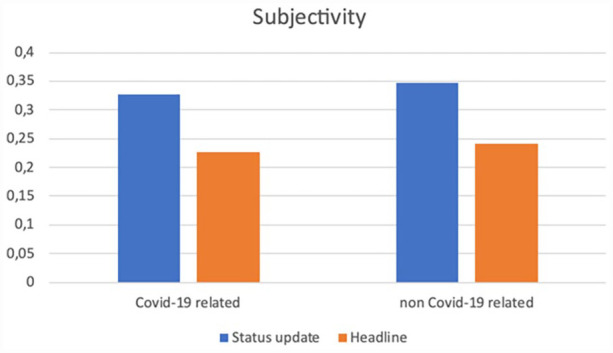
Subjectivity of online articles’ headlines and Facebook status updates.

**Figure 3.**
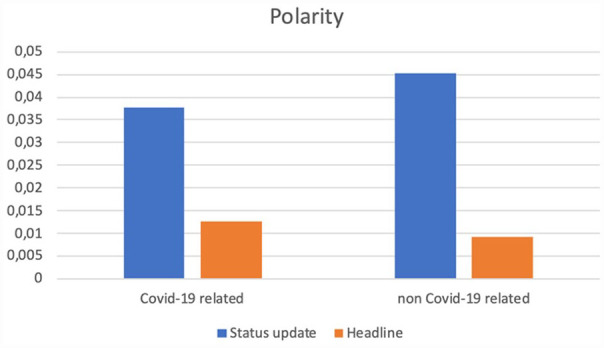
Polarity of online articles’ headlines and Facebook status updates.

Before we zoom in on how social media editors (SMEs) packaged the news in the status message on top of the Facebook news post (see Appendix B for a visual example), we compare the subjectivity and polarity between the status update and the actual headline of the article (Figures 2 and 3).

We indeed find that the subjectivity of the status messages (*M* = 0.34; SD = 0.35), is higher compared to subjectivity in the accompanying headline of the article (*M* = 0.24; SD = 0.33), *t*(140,358) = 85.765, *p* < 0.000, 95% CI [0.101, 0.106]). A similar result was found for polarity, which is on average higher in the status update (*M* = 0.04; SD = 0.26) than in the headline of the article (*M* = 0.01; SD = 0.22), *t*(140,358) = 37.235, *p* < 0.000, 95% CI [0.030, 0.034]). Thus, as expected based on the different logics that apply on social media sites (e.g. van Dijck and Poell, 2013; Welbers and Opgenhaffen, 2019), the articles are packaged in a status update with higher subjectivity and polarity scores than the actual news article headline.

### COVID-19-related versus other types of news

Next we zoom in on the differences between these two parameters among headlines and Facebook status updates of articles that are either related to COVID-19 or not at all. Regarding subjectivity, we see that headlines and status updates of COVID-related news items carry less subjectivity than those of non-COVID-related posts. In the case of polarity, we see that headlines of COVID news use slightly more positive wording than those of non-COVID news, although both scores are situated around zero and can thus be considered fairly neutral. In the case of status updates, we see somewhat more polarity, where we can also observe that more positive wording is used in non-pandemic-related news than in the case of reports on COVID, which is in line with what we observed earlier about subjectivity.

Overall, we find that status updates as written by SMEs to accompany and ‘sell’ online news articles score considerably higher on both polarity and subjectivity when compared to the articles’ headlines, which are in most cases chosen by (online) journalists. If we look at the overall subjectivity of only Facebook status messages accompanying COVID-19-related news articles (*M* = 0.33; SD = 0.33) and compare it with that of non-COVID-19-related messages (*M* = 0.35; SD = 0.35), *t*(109,962) = −11.519, *p* < 0.000, 95% CI [−0.025, −0.0179], we see that the subjectivity for non-pandemic-related captions is slightly higher. For polarity, COVID-19 related statuses (*M* = 0.04; SD = 0.24) score on average lower on polarity than non-COVID-19 messages (*M* = 0.05; SD = 0.28), *t*(117,327) = −5.34, *p* < 0.000, 95%, CI [−0.010, −0.005]. Both subjectivity and polarity are thus higher for Facebook status messages accompanying non-COVID-19 related news than for COVID-19 related news. These findings nuance and remonstrate the assumption that Facebook status updates are more prone to expressing higher degrees of subjectivity or polarity by default. It seems that in line with previous insights (Hågvar, 2019; Opgenhaffen, 2021) when reporting on COVID in Facebook messages, journalists and SMES are somewhat more reluctant to engage in subjectivity and polarity in their use of language due to the delicacy of the topic. We consider this a relevant finding for international (future) scholarship on the work and output of social media editors packaging online news.

**Figure 4.**
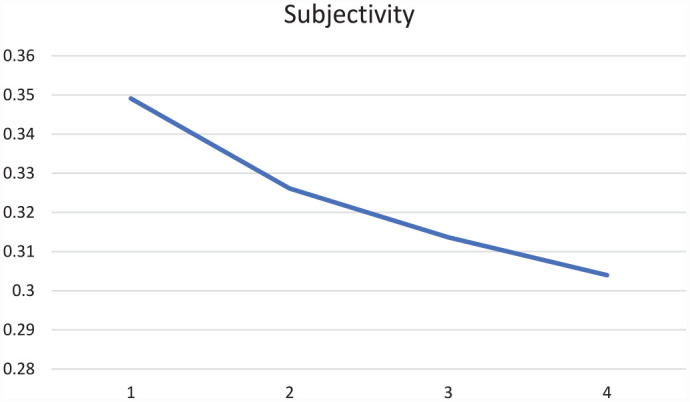
Subjectivity in Facebook status messages over time.

**Figure 5.**
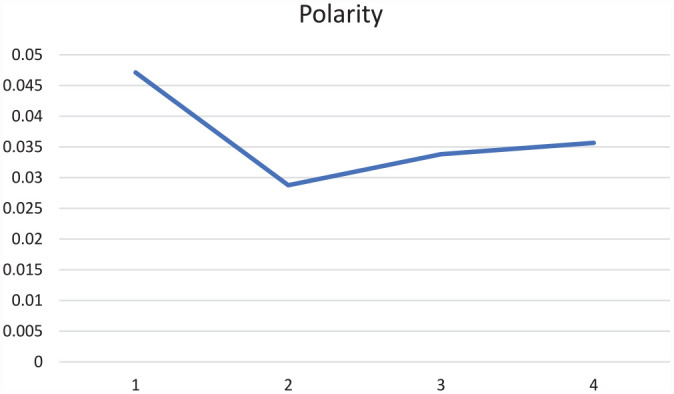
Polarity in Facebook status messages over time.

Throughout the four periods within our time of study, overall subjectivity of the 50,350 COVID-related news messages’ status updates gradually declined. At the onset of the pandemic, thus in Period 1, the overall subjectivity was notably higher, although its score peaked at 0.349 (on a scale from 0 to 1) and thus we find that subjectivity overall remained rather moderate. For polarity, we see a less clear trend with a drop in the second period but apart from that similar averages for polarity scores. Note that these scores are measured from −1 to 1, meaning on the one hand that the overall measured polarity is more positive than it is negative. On the other hand, the polarity scores are quite close to 0; a score of 0 equals neutrality of a Facebook post (Figures 4 and 5).

To gain an even better understanding of the use of subjectivity and polarity in status messages and the difference between COVID versus Non-COVID news, we identified some distinct clusters of status messages. Using a cluster analysis and the TwoStep algorithm (Average Silhouette = 0.7), we retrieve four distinct clusters of status messages following their respective polarity and subjectivity as calculated automatically. The first cluster comprises neutral messages, which together constitute 47.8% of our total dataset. They score −0.00 for polarity and just 0.02 for subjectivity. This means that nearly half of all assessed status messages of Flemish news outlets are neutral in nature. The second largest group consists of messages that are subjective (0.49) yet do not hold a high level of polarity (0.07), which counts for 28.5% of our total data set. Just 23.7% of our data set, or just over 33,000 posts, had status messages which were very subjective and outspokenly positive or negative. In the former group, polarity scored 0.53 and subjectivity 0.81 – in the latter −0.40 and 0.77. This means that outspokenly positive messages are slightly more subjective than negative messages, while the ratio between them separately is 12.5% and 11.2% of our total sample.

When calculating the shares of our four clusters for news posts which do (not) relate to COVID-19 news reports (see Table 1), a few notable differences surface. Percentagewise, Facebook status messages accompanying pandemic-related news are both less positive and negative when compared with messages of other news types, although the difference between positivity is more pronounced. Our dataset of COVID-19-related status messages were at the same time less subjective.

When further differentiating the above findings for COVID-19 status messages per quarter, we see that the cluster of neutral messages increased throughout the year. In March–May 2020 this share was still 32.5% and that grew steadily throughout the year to 36.7% in December 2020–March 2021. At the same time, we see that the share of messages that are simply or distinctly subjective and emphasize positivity has remained fairly stable. For example, we see a stabilization for the cluster of subjective messages with low polarity from 21,1% in the first period and 22,3% in the fourth period, and also a stabilization of the cluster labelled as very subjective, outspoken positive. Interestingly, the negative polarity seems to decrease throughout the corona year, with a share of 24,8% for the cluster very subjective combined with outspoken negative in the first period, which decreased to 21.1% towards the last quarter. In other words, status messages of Flemish news brands’ Facebook news posts dealing with COVID-19 became progressively more neutral and less outspoken negative over time. We infer that the coverage ‘normalised’ over time after the pandemic had become no longer a novel thing but a seemingly permanent fixture in people’s daily lives.

### Mainstream versus alternative media

When we divide the news media according to mainstream vs. alternative media, we can map whether there are differences in terms of how they packaged their corona-related messages via the status messages. The results show that the alternative news media (*M* = 0.37, SD = 0.31) injected significantly more subjectivity into their posts than the more mainstream titles (*M* = 0.31, SD = 0.33), *t*(9833.3) = −13.23, *p* < 0.001). For polarity we saw no difference, with a degree of polarity of *M* = 0.04, SD = 0.24 for the mainstream media and *M* = 0.035, SD = 0.23 (*t*(9640.5) = 0.93, *p* = 0.35) for the alternative media. When we zoom in to only the alternative media and distinguish between the three self-proclaimed left-wing news media and the three rather right-wing media, we again see no difference in terms of the degree of polarized language use (*M* = 0.036, SD = vs *M* = 0.032, *t*(7049.9) = −0.30, *p* = 0.77), but a clear and significant difference in terms of subjectivity. The degree of subjectivity in the status messages of the left-wing outlets is on average *M* = 0.36, SD = 0.30, indicating a slightly less subjective discourse compared to the three right-wing news titles (*M* = 0.38, SD = 0.32), *t*(7022.3) = 2.99, *p* = 0.002. Moreover, the degree of subjectivity in these status messages differs from the way they shape their more conventional headline (i.e. the one on the online news site), which is *M* = 0.21, SD = 0.31 for the left-wing news media and *M* = 0.21, SD = 0.30 for the right-wing media. Subjectivity is more freely used in status messages.

### Use of emojis and linguistic features

Our final analysis reveals that 13.01% of all news posts not related to the COVID-19 pandemic are accompanied by at least one emoji. For pandemic-related posts, this is only 9.9%, which is significantly lower. This is in line with Hågvar’s (2019) and Opgenhaffen’s (2021) notion of a specific dynamic when it comes to using emojis in Facebook news posts: the degree of controversy of a story is inversely proportional to the likelihood of emojis. Also in line with this is the notable increase in emoji use by Flemish SMEs for COVID-19-related news posts throughout our four distinguished quarters. The percentage share rose progressively over time from 8.9% to 9.5%, 10.4% and finally 11.5% of the COVID-19-related posts. This is not a surprise as the diminished novelty of the pandemic clearly spurred different approaches in handling it from a newsroom’s perspective, including packaging news stories via social media platforms.

Vice versa, we see patterns of decrease for the use of question marks in Facebook captions. They are more recurring in pandemic-related reporting (10.31% of all related captions) than in other news topics (9.6%) and specifically for the former category, were more dominant in the first quarter (March–May 2020, 11.61%) than in the other three (10.2%, 9.3% and 9.4%, respectively). The use of question marks as a visual cue to stimulate audience engagement can be understood as an additional means to reflect and engage with possible questions citizens had in the first months of the pandemic, and to boost user interaction.

Exclamation marks, on the other hand, were far less recurrent in news captions accompanying pandemic-related news articles than those dealing with different subjects (2.9% vs 5.6% of the respective total *n*’s). Just as with question marks, they were used more often in the first quarter as opposed to the three following ones, with shares for COVID-19-related captions diminishing from 4.0 to 2.5, 2.1 and 2.2.

When we zoom in on the mainstream vs. alternative news media conundrum, specifically regarding the use of emoji and linguistic features in COVID related news, it yields some interesting findings. For example, alternative media appear to use emoji in their status messages more often than more mainstream media (20.8% vs 8.2%) and are also more likely to use a question mark (13.6% vs 9.8%) and exclamation point to reinforce their message (4.5% vs 2.6%). When we look at the difference between alternative news media among themselves and we analyse only the titles that adhere to a clear ideology, we see that the right-wing news media (36.3%) use emoji in their status messages remarkably more than left-wing media (2.8%) in their COVID-19 related posts. They also make more use of exclamation points (7.1%) than their counterparts (1.6%). Only when it comes to the use of question marks, we see that left-wing titles (16.4%) do so more often than right-wing media (11.22%). Based on this, we could argue that alternative media seem to be more daring to experiment with social media rhetoric than more traditional media when covering COVID-19, and that this is certainly true for right-wing titles.

## Discussion and conclusions

Our analyses yielded a range of findings, each shining light on the thus far much under-researched output of social media editors (SMEs), who repeatedly use emotional, surprising and/or subjective story elements in the status messages accompanying hyperlinks to news outlets’ online articles (Lischka, 2021; Tsuriel et al., 2021) and with the marketing-driven aim to ‘sell’ articles and both online audience engagement and subscription rates using social media logic (van Dijck and Poell, 2013). One of our key findings is that these status messages score significantly higher than traditional news article headlines for degrees of polarity and subjectivity alike. This is a logical finding due to the different nature of a headline and an extra message intended to convince people to consume content but is here empirically proven for one of the first times and in correspondence with existing scholarship (Welbers and Opgenhaffen, 2019).

As an additional layer of our research and contribution to academia, we differentiated to answer the first research question between Facebook news posts which do and do not deal with reporting on the COVID-19 pandemic. Summarising our key insights, we find that about a third of all news posts assessed are linked to pandemic-related news reporting. In status messages both subjectivity and polarity are lower for COVID-19-related news than for news posts not dealing with the pandemic. Polarity, as well as the use of question marks and interrogation marks in pandemic-related news posts was highest in the first quarter of our analysis (March–May 2020), before cooling down afterwards. In the case of the question marks, this probably has to do with the presence of uncertainty in the early days that was translated through the status messages, while the use of exclamation marks, in turn, is prevalent with the breaking news content in the outbreak of the pandemic. The opposite applies for the use of emojis whose use evidently increased throughout the first year of the pandemic, which is in line with what Hågvar (2019) stated about the relationship between the use of emoji and the controversy of the news topic. We contextualise these findings by assuming that the pandemic had its peak in terms of novelty, impact on public life and newsworthiness (thus far) in the early months of strict lockdowns and other restrictions. This was already linked to increases in general news consumption in those first months of the pandemic (Newman et al., 2021), where a similar cooling down-process became apparent afterwards. As COVID-19 became a fixture in daily life and public debate, attention for the topic among the population started to wane, which is clearly also reflected in the normalising aspects of our study.

This study corresponds to previous scholarship on the tasks and role of social media editors (SMEs) within contemporary newsrooms (Lischka, 2021; Opgenhaffen, 2021; Tsuriel et al., 2021), although from an empirical perspective. We contextualise our findings within the existing body of literature by gauging that SMEs even in times of crises, SMES have continued their work of packaging and selling news via status updates. Indeed, we saw that in line of previous research, status messages were more subjective and polarized than the more traditional headlines. However, this research also shows that SMEs take into account the controversy and the delicacy of the topic, generally applying fewer rhetorical tricks in status messages around corona news than in non-COVID news, which is also a confirmation of previous research. Scholarly attention for SMEs and their work is rightfully increasing as they will inevitably remain vital in maintaining online audience engagement. Additional scholarship, however, could look deeper into the distinct function of SMEs within newsrooms from an ethnographic perspective, as to better pinpoint their exact duties and responsibilities both within newsrooms and media companies, but also within society as drivers on informing citizens and shaping and maintaining trust in journalism as the fourth estate. A more conceptual angle in particular is desired; as a starting point, we position SMEs at the crossroads of production, content and consumption diversity and invite future scholars to address SMEs from a news diversity framework.

Our study specifically dealt with mainstream and alternative outlets from Flanders (Belgium), a distinct media market with its own media system, regulatory framework and news outlets. To answer our second research question, we found that Facebook status messages of alternative outlets are more subjective than those of their mainstream counterparts, but not necessarily using more positive or negative wordings. Notably right-skewing alternative outlets use linguistical features such as emoji, question and interrogation marks far more often, and thus seem to be daring to experiment more with a social media rhetoric, probably to differentiate themselves from the more mainstream media. These findings complement and contribute to other recent findings on the social media behaviour of (right-wing) alternative news media (Boberg et al., 2020; Hiaeshutter-Rice & Weeks, 2021). Although we cannot completely guarantee transferability of our findings to other regions and/or markets, we still gauge that similar conclusions will be reached elsewhere as well. In this paper, we combined the use of social media as gateways for online news and reporting on the COVID-19 pandemic. It goes without saying that both aspects are prime examples of joint features as part of a rapidly digitised and internationalised society with similar characteristics, issues and questions to be answered.

On a final note, we acknowledge a few shortcomings. We deductively differentiated between mainstream and alternative news outlets, while an inductive approach could have been equally relevant and could have yielded slightly different results. We were also limited in the number of news outlets as we only looked at the small media market of Flanders (Belgium), whereas studies from larger and/or several markets and their outlets could provide more (detailed) findings. We also acknowledge our analysis on the use of emoji and linguistic features being rather rudimentary. Future research is welcome to delve deeper in the relationship between visual cues and audience engagement on platforms such as Facebook, for instance by looking if the use of several emoji further affects engagement either positively or negatively. Finally, although the used lexicon approach is an effective tool to study subjectivity and polarity, certain limitations should be considered. The lexicon approach was trained on book reviews and Facebook posts (and headlines) are generally much shorter, which might affect the reliability of the used method. Another limitation of the approach is that we only studied the macro level and did not go deeper into the detailed content of the Facebook posts. For a deeper investigation of sentiment, more advanced techniques or human coding is required.

## Figures and Tables

**Figure B1.**
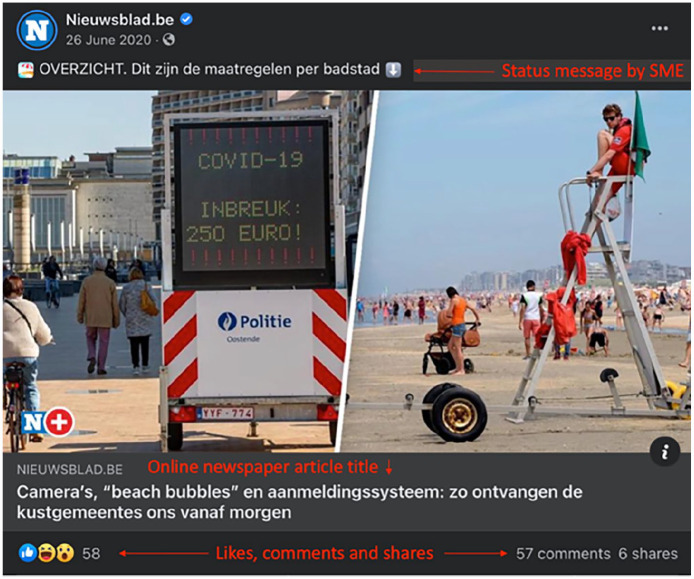
An example of a COVID-19-related news post by Nieuwsblad.be, Flanders’ second biggest print and online news brand. The caption as written by the social media editor translates as ‘OVERVIEW’. These are the measures per coastal town’ and uses emojis to incite readers clicking through to the online article on the Nieuwsblad.be website.

## Appendix A: Keywords

**Table A1.** Keywords in Dutch and translated to English.

Dutch keywords	Translated keywords
1.5 m	1.5 m
Afstand	Distance
Besmettingen	Infections
Bubbel	Bubble
Corona	–
Coronapandemie	Corona pandemic
Coronavirus	–
COVID	–
COVID-19	–
ICU	–
Intensive zorg	Intensive care
Intentieve zorgen	Intensive care
Lockdown	–
Maatregelen	Measures
Mondkapje	Face mask
Mondmasker	Face mask
Opnames	Hospitalisations
Overlegcomité*	Conciliation committee
Overlijdens	Deaths
Pandemie	Pandemic
SARS	–
Vaccin	Vaccine
Vaccinatie	Vaccination
Variant	–
Veiligheidsraad*	Safety council
Versoepelingen	Relaxations
Verstrengingen	Hardening, escalations
Virologen	Virologists
Viroloog	Virologist
Virus	–
Ziekenhuisopnames	Hospitalisations

The terms *overlegcomité* and *veiligheidsraad* are both used interchangeably for the meetings of various Belgian governments to decide on relaxing or tightening existing COVID-19 measures.

* obviously.
